# Renal proximal tubulopathy in an HIV-infected patient treated with tenofovir alafenamide and gentamicin: a case report

**DOI:** 10.1186/s12882-020-01981-9

**Published:** 2020-08-12

**Authors:** Jack E. Heron, Mark Bloch, Vinay Vanguru, John Saunders, David M. Gracey

**Affiliations:** 1 Department of Renal Medicine, Royal Prince Alfred Hospital, Camperdown, New South Wales Australia; 2 Holdsworth House Medical Practice, Sydney, New South Wales Australia; 3 The Kirby Institute, University of New South Wales, Sydney, New South Wales Australia; 4 Department of Haematology, Royal Prince Alfred Hospital, Camperdown, New South Wales Australia; 5 Central Clinical School, The University of Sydney, Sydney, New South Wales Australia

**Keywords:** Fanconi syndrome, Tenofovir alafenamide, Antiretroviral therapy, Nephrotoxicity, Case report

## Abstract

**Background:**

The nucleotide reverse transcriptase inhibitor Tenofovir Alafenamide (TAF) is a novel pro-drug of tenofovir (TFV) and possesses a superior renal safety profile compared with tenofovir disoproxil fumerate (TDF). Due to unique pharmacokinetic characteristics, treatment with TAF is not associated with significant renal proximal tubular accumulation of TFV. TAF is associated with a lower risk of acute kidney injury, chronic kidney disease, proteinuria and renal proximal tubular dysfunction than treatment with TDF. No cases of Fanconi syndrome have been reported in clinical trials of TAF. It is unknown whether treatment with TAF can lead to accumulation of TFV in proximal tubular cells and cause nephrotoxicity under certain clinical circumstances.

**Case presentation:**

Here we report the case of a patient on stable TAF-based antiretroviral therapy with for HIV-1 infection who developed proximal tubulopathy when treated with gentamicin for febrile neutropenia in the context of relapsed Hodgkin lymphoma. Eighteen days after commencing chemotherapy for relapsed Hodgkin lymphoma the patient presented to hospital with fevers, hypotension and neutropenia. The patient was commenced on piperacillin, tazobactam and gentamicin. Within 24 h the patient developed marked hypokalaemia and hypophosphataemia requiring intravenous replacement therapy. There was proteinuria, glycosuria and evidence of marked urinary electrolyte wasting, consistent with acute proximal tubular dysfunction. Eleven days after the gentamicin was stopped the serum biochemistry normalised. The urinary electrolyte wasting and proteinuria had improved, and the glycosuria had resolved.

**Conclusion:**

This is the first case report to describe acute renal proximal tubulopathy in an HIV-infected patient treated with TAF and gentamicin. As the number of patients prescribed TAF outside the clinical trial setting increases, so too does the potential for previously unreported drug interactions and adverse events. Clinicians need to be aware of potential unreported adverse drug reactions as the use of TAF becomes increasingly common in clinical practice.

## Background

The nucleotide reverse transcriptase inhibitor, Tenofovir Alafenamide (TAF), is a novel pro-drug of the active metabolite tenofovir (TFV), which is effective for the treatment and prevention of HIV-1 infection, as well as the treatment of chronic hepatitis B. It has a superior renal and bone safety profile when compared with its sister drug, Tenofovir Disoproxil Fumerate (TDF) [1]. In contrast to TDF, treatment with TAF does not result in the accumulation of high concentrations of TFV in renal proximal tubular cells [2]. TAF undergoes the majority of its conversion to TFV in peripherally circulating blood lymphocytes, resulting in higher TFV levels within HIV-infected cells and lower plasma levels when compared to orally administered TDF [2]. The use of TAF is associated with a lower risk of acute kidney injury, chronic kidney disease, renal proximal tubular dysfunction and the Fanconi syndrome, compared to TDF [1, 3, 4]. TAF appears to be safe in patients with significantly impaired renal function and has been used in patients on haemodialysis [5]. Despite this, a possible case of TAF-associated mitochondrial dysfunction [6] and a case of acute kidney injury with the switch to TAF have been reported [7]. It is unclear whether, under specific clinical conditions, treatment with TAF can lead to accumulation of TFV in proximal tubular cells and cause nephrotoxicity [8]. A number of other drugs may also induce renal tubular dysfunction including cisplatin, cidofovir, gentamicin, azathioprine, valproic acid and ranitidine [9]. Pharmacological synergism between TAF and these other tubular toxins, producing significant proximal tubular dysfunction, has not been reported.

## Case presentation

Here we report the case of a 46-year-old patient with a 17-year history of HIV infection on stable antiretroviral therapy (ART) with TAF 10 mg orally daily, emtricitabine, darunivir and cobicistat who developed renal proximal tubulopathy in the setting of gentamicin therapy for febrile neutropenia complicating the treatment of relapsed Hodgkin lymphoma.

The patient had been diagnosed with Hodgkin lymphoma in 2011 and achieved a complete disease remission following treatment with doxorubicin, bleomycin, vinblastine and dacarbazine (ABVD). In July 2019 he presented with febrile neutropenia and anaemia, and underwent a positron emission tomography scan and bone marrow biopsy, which confirmed a relapse of Hodgkin lymphoma. He was commenced on dexamethasone, cytarabine and carboplatin (DHAC).

In addition to HIV-infection, he had a past medical history of mucocutaneous herpes simplex virus, cutaneous herpes zoster, cervical spondylopathy and hepatitis C virus infection with spontaneous viral clearance. The patient's previous ART included TDF/emtricitabine and ritonavir boosted lopinavir. The current HIV viral load was low-positive. In addition to his ART, the other regular medications included valaciclovir, pantoprazole, olanzapine, fluconazole, citalopram, prn oxycodone and prn paracetamol.

Blood chemistry prior to commencing DHAC was unremarkable with a normal serum potassium, phosphate and bicarbonate concentration. The serum biochemical parameters had remained normal until the patient was admitted to hospital with febrile neutropenia 18 days after commencing chemotherapy. On presentation to hospital the patient’s white cell count was 0.7 × 10^9^/L, with a lymphocyte count of 0.0 × 10^9^/L. The serum creatinine was 82 μmol/L (Reference range: 60-110 μmol/L) with an estimated glomerular filtration rate (eGFR) of greater than 90 ml/min/1.73m^2^. The serum potassium was 3.9mmom/L (Reference range: 3.5–5.2 mmol/L) and serum phosphate was 0.89 mmol/L (0.75–1.5 mmol/L). The patient was commenced on piperacillin, tazobactam and gentamicin for febrile neutropenia. The source of sepsis was unclear. The patient received two daily doses of gentamicin at 3.8 mg/kg, and a third dose on day four of the admission of 5.8 mg/kg in the context of sepsis and persistent hypotension.

Within 24 h of receiving gentamicin the patient developed marked hypophosphatemia and hypokalemia, which was difficult to correct despite oral and intravenous replacement, as shown in Table 1. The serum bicarbonate remained normal. Despite the presence of hypokalaemia and hypophosphataemia, the urinary potassium concentration was 24 mmol/L with a fractional excretion of 21.5%, and the urinary phosphate concentration was 8 mmol/L with a fractional excretion of 60.4%. There was proteinuria, with a urinary protein creatinine ratio of 162.5 mg/mmol creatinine (Reference range: < 12 mg/mmol creatinine). There was glycosuria, with a urine glucose concentration of 107 mmol/L, however, in the context of a blood glucose concentration of 18 mmol/L. The hypophosphataemia persisted for eleven days and the hypokalaemia persisted for seven days. Clinically, the picture was that of renal proximal tubulopathy; the gentamicin was discontinued when proximal tubulopathy was noted. Testing for hyperaminoaciduria and other markers of proximal tubular dysfunction was not performed.

**Table 1:** Laboratory results. The days listed are in reference to the date of admission, which was also the date of first gentamicin exposure

	Pre-admission	Day 0 16/9	Day 1	Day 2	Day 4	Day 5	Day 7	Day 9	Day 11	Day 13	Day 14	Day 16
Serum creatinine (μmol/L) (Reference range: 60-110)	84	82	69	69	67	73	80	66	72	72	84	74
eGFR (mL/min/1.73m^2^) (Reference range: >60)	> 90	> 90	> 90	> 90	> 90	> 90	> 90	> 90	> 90	> 90	> 90	> 90
Serum potassium (mmol/L) (Reference range: 3.5-5.2)	4.2	3.9	↓ 3.0	↓ 3.0	↓ 3.1	↓ 3.3	4.2	4.6	4.6	4.7	4.3	4.3
Serum chloride (mmol/L) (Reference range: 95-110)	↓90	↓87	↓88	↓93	↓87	↓87	↓90	↓88	↓ 90	95	↓ 94	96
Serum phosphate (mmol/L) (Reference range: 0.75-1.5)	0.96	0.89	↓ 0.49	↓ 0.67	↓ 0.47	↓ 0.72	↓ 0.53	↓ 0.59	↓ 0.54	1.0	1.05	1.14
Serum magnesium (mmol/L) (Reference range: 0.7-1.1)	0.77	0.60	↓0.51	↓0.52	0.84	↓0.62	↓0.69	0.87	0.71	0.74	↓0.65	0.70
Urine potassium (mmol/L)					24							40
Fractional excretion of potassium					↑ 21.5%							↑ 24.6%
Urine phosphate (mmol/L)							8.0					6
Fractional excretion of phosphate							↑ 60.5%					13.9%
Urine glucose (mmol/L)								↑ 107			↑ 4.2	0.2
Urine protein creatinine ratio (mg/mmol creatinine) (Reference range: <12)					↑ 154			↑ 162				↑ 39.3

Eleven days after the last dose of gentamicin, and following normalisation of the serum biochemistry, the urine biochemistry was repeated (Table 1). The urinary potassium concentration was 40 mmol/L, with a fractional excretion of 24.6%. The urinary phosphate concentration was 6 mmol/L, with a fractional excretion of 13.9%. The urine glucose concentration was 0.2 mmol/L. The urine protein creatinine ratio had fallen to 39.3 mg/mmol creatinine. Consideration was given to stopping the TAF, however, in view of the patient’s biochemical improvement it was elected to continue this, with close monitoring. The patient continued treatment for relapsed Hodgkin lymphoma without recurrence of proximal tubulopathy.

## Discussion and conclusion

This is the first case report to describe renal proximal tubulopathy in an HIV-infected patient treated with TAF related to concomitant exposure to gentamicin. The patient had acute onset of marked urinary wasting of phosphate, potassium and glucose. Under normal conditions phosphate is freely filtered at the glomerulus and 60 to 70% is reabsorbed in the proximal tubule [10]. A fractional excretion of 60.4%, in the setting of hypophosphatemia, is strongly suggestive of proximal tubular dysfunction. Similarly, under normal conditions the presence of hypokalaemia is associated with a marked reduction in the fractional excretion of potassium, usually to less than 2.8% [11]. In our case the fractional excretion of potassium was greater than 20%. Transient glycosuria and proteinuria were also noted.

Aminoglycoside-induced proximal tubular dysfunction is well described. In all previously reported cases, proximal tubular dysfunction has occurred in the setting of prolonged high dose aminoglycoside exposure. In a total of eight reported cases the duration of drug exposure before the onset of clinically apparent proximal tubulopathy has ranged from six to 33 days [12–16]. In contrast, our patient received only three doses, with evidence of tubulopathy after the first dose. Carboplatin was also administered in this case but is only associated with subclinical proximal tubular dysfunction [17]; we are not aware of any published cases of Fanconi syndrome primarily attributable to carboplatin.

The proximal tubular dysfunction associated with TFV is well characterised and includes non-glomerular proteinuria, acute tubular necrosis, nephrogenic diabetes insipidus and by multiple proximal tubular transport defects leading to urinary wasting of glucose, phosphate, potassium, bicarbonate, uric acid, amino acids and other solutes (known as Fanconi syndrome) [18]. TDF and TAF are both prodrugs of TFV. TDF is rapidly metabolised to TFV in plasma leading to high plasma concentrations. TFV is a substrate for organic anion transporter 1 (OAT-1) found in the proximal tubular basolateral membrane. TFV accumulates in proximal tubular cells where concentrations far exceed those in blood or peripherally circulating blood lymphocytes [19]. TFV causes mitochondrial toxicity in proximal tubular cells, likely through depletion of mitochondrial DNA, leading to failure of ATP production, cellular dysfunction and/or death [8, 20]. In contrast to TDF, TAF is not significantly metabolised to TFV in plasma. TAF is metabolised to TFV intracellularly by Cathepsin A, a hydrolase predominantly expressed in lymphoid cells, but also in other tissue including the kidney and liver [21]. These pharmacokinetic characteristics of TAF result in higher TFV concentrations in peripherally circulating blood lymphocytes at lower oral doses and with lower plasma concentrations than TDF [22]. Also, TAF itself does not appear to be a substrate for renal organic anion transporters, the main transporters responsible for the uptake and accumulation of TFV in proximal tubular cells [23].

The mechanisms of drug-induced proximal tubular dysfunction are poorly understood and include mitochondrial dysfunction, glutathione depletion, failure of sodium-coupled active solute transport driven by Na^+^-K^+^-ATPase, apical membrane dysfunction, brush border disruption and impaired lyposomal enzyme activity [18, 24, 25]. Mitochondrial dysfunction is implicated in both aminoglycoside and tenofovir-associated nephrotoxicty, raising the possibility of a synergistic mitochondrial toxicity in our case [20] . It is unknown whether, under certain clinical circumstances, TAF administration can lead to the accumulation of TFV in proximal tubular cells and induce nephrotoxicty [8]. Histological evidence of mitochondrial dysfunction has been reported in a patient receiving TAF, however, in the context of multiple other renal lesions including diabetic nephropathy and immune complex deposition [6]. A number of biomarkers have been investigated in the setting of proximal tubular dysfunction, including low-molecular weight proteins that are freely filtered at the glomerulus and, under normal circumstances, reabsorbed and metabolised in the proximal tubule. Urinary beta-2-microglobulin appears to be a sensitive biomarker of proximal tubular dysfunction in TDF-treated patients [26]. Urinary retinol-binding protein appears to be most specific for mitochondrial dysfunction as its tubular reabsorption is highly ATP dependent [26]. Other biomarkers, including N-acetyl-β-D-glucosaminidase, may allow proximal tubular cellular dysfunction to be distinguished from cellular damage or death [26]. These biomarkers were not performed during the investigation of this case but may be informative in confirming the presence and pathophysiology of tubular dysfunction when it is suspected. Drug synergism in the pathophysiology of proximal tubular dysfunction has been described; including between cisplatin and ifosfamide [27], and between cefazolin, TDF and aminoglycosides [15, 28]. Of note, the uptake of some renal tubular toxins is upregulated in the setting of sepsis and renal ischaemia [29]. Whether this is the case for TFV or TAF is unknown. The impact of absolute lymphopenia and lymphoid malignancy on the metabolism of TAF is also unknown, but may reduce the proportion of TAF metabolised intracellularly by Cathepsin A, thereby increasing plasma concentrations and the risk of proximal tubular accumulation.

While we cannot definitively conclude an association between TAF and proximal tubulopathy based on this case alone, we hypothesise that sepsis and lymphopenia led to the accumulation of both TFV and gentamicin in proximal tubular cells where they acted synergistically to cause mitochondrial toxicity and proximal tubular dysfunction. Patients are commonly switched from TDF to TAF-based antiretroviral regimens on the basis of a superior side effect profile, and in particular, because of a lower rate of renal adverse events reported in clinical trials [1, 4]. As the number of patients prescribed TAF outside the clinical trial setting increases, so too does the potential for unreported drug interactions and adverse events. Clinicians need to be aware of potential unreported adverse drug reactions as the use of TAF becomes increasingly common in clinical practice.

## Data Availability

Not applicable.

## Abbreviations

ABVD: Doxorubicin, bleomycin, vinblastine and dacarbazine
ART: Antiretroviral therapy
DHAC: Dexamethasone, cytarabine and carboplatin
eGFR: Estimated glomerular filtration rate
OAT-1: Organic anion transporter 1
TAF: Tenofovir alafenamide
TDF: Tenofovir disoproxil fumerate
TFV: Tenofovir
